# A Nested PCR Telomere Fusion Assay Highlights the Widespread End-Capping Protection of Arabidopsis CTC1

**DOI:** 10.3390/ijms25010672

**Published:** 2024-01-04

**Authors:** María I. Vaquero-Sedas, Miguel A. Vega-Palas

**Affiliations:** Instituto de Bioquímica Vegetal y Fotosíntesis, CSIC-Universidad de Sevilla, IBVF (CSIC-US), E41092 Seville, Spain; m.isabel@ibvf.csic.es

**Keywords:** *Arabidopsis thaliana*, telomere capping, telomere fusions analysis, genome stability, CST complex, CTC1

## Abstract

Telomeres protect the ends of linear eukaryotic chromosomes from being recognized as DNA double-strand breaks. Two major protein complexes are involved in the protection of telomeres: shelterin and CST. The dysfunction of these complexes can challenge the function of telomeres and lead to telomere fusions, breakage–fusion–bridge cycles, and cell death. Therefore, monitoring telomere fusions helps to understand telomeres biology. Telomere fusions are often analyzed by Fluorescent In Situ Hybridization (FISH) or PCR. Usually, both methods involve hybridization with a telomeric probe, which allows the detection of fusions containing telomeric sequences, but not of those lacking them. With the aim of detecting both types of fusion events, we have developed a nested PCR method to analyze telomere fusions in *Arabidopsis thaliana*. This method is simple, accurate, and does not require hybridization. We have used it to analyze telomere fusions in wild-type and mutant plants altered in CTC1, one of the three components of the Arabidopsis CST telomere capping complex. Our results show that null *ctc1-2* mutant plants display fusions between all telomeric regions present in Arabidopsis chromosomes 1, 3 and 5, thus highlighting the widespread end-capping protection achieved by CTC1.

## 1. Introduction

Telomeres localize at the ends of eukaryotic chromosomes and usually contain G/C-rich tandem arrays of double-strand telomeric repeats followed by an extension of the G-rich strand at the 3′ end. These double and single-strand repeats associate with multiple proteins, giving rise to nucleoprotein complexes that solve two fundamental problems. On the one hand, they solve the replication problem that arises as a consequence of the limitation that regular DNA polymerases have to synthesize the lagging strand at the end of linear DNA molecules. On the other hand, they solve the protection problem that arises at DNA ends and double-strand breaks [1]. The end-replication problem is usually solved by the enzyme telomerase, which is a retrotranscriptase that adds telomeric repeats to the 3′ chromosome ends by using an RNA template, and by additional proteins that also contribute to filling in the 3′ end extended by telomerase. In addition, the end-replication problem can also be addressed through a recombination mechanism termed Alternative Lengthening of Telomeres (ALT). With regard to the end-protection problem, two conserved protein complexes contribute to bypassing it by preventing the end of the chromosomes from being recognized as DNA breaks. These capping complexes, which are known as shelterin and CST, also contribute to solving the end-replication problem [2,3,4,5].

In mammals, the shelterin complex includes TRF1, TRF2, POT1, TIN2, TPP1 and RAP1. Whereas TRF1 and TRF2 bind to the double-strand telomeric repeats, POT1 binds to the single-strand overhang. These three proteins, together with RAP1, which binds to TRF2, and TIN2-TPP1, which bridge TRF1 and TRF2 with POT1, cap telomeres, keeping them in a closed protected state that is refractory to DNA damage sensing and repair. Similarly, the mammal CST complex, which includes the proteins CTC1, STN1 and TEN1, binds to single-strand telomeric DNA through CTC1, caps telomeres, and prevents sensing and repair of DNA. When the integrities of the shelterin or the CST complexes are compromised, the DNA damage response (DDR) machinery recognizes telomeres as broken ends and process them, leading to different types of recombination events, including inter-telomeric recombination. Inter-telomeric recombination events can generate telomere fusions, which are very harmful for the cell because they create dicentric chromosomes. These dicentric chromosomes break in anaphase, leading to unstable chromosomes that can fuse during the next mitosis, giving rise to a catastrophic breakage–fusion–bridge cycle [2,3]. Therefore, the study of telomere fusions informs on different aspects of telomere functions and constitutes a fundamental tool for the study of telomere biology.

Interestingly, some components of the DDR machinery can also associate with mammal telomeres and contribute to capping them. This is the case for the KU70/KU80 heterodimer, which participates in the classical nonhomologous end-joining pathway at genome-wide level. Although this heterodimer contributes to the fusion of telomeres in cells with mutated capping proteins like TRF2, it is also involved in the protection of telomeres in wild-type (WT) cells. Indeed, mouse mutants altered only in KU70 or KU80 exhibit telomere fusions [2,6,7]. Thus, the capping of mammal telomeres involves a complex interplay between proteins that protect them and the DDR machinery.

In Arabidopsis, telomeres consist of tandem arrays of the plant-type telomeric repeat unit (CCCTAAA/TTTAGGG) that spread 2.5–5 Kbp. The very ends of Arabidopsis telomeres are quite unique. Whereas half of them are blunt, the other half contain the canonical 3′ overhang of the G-rich strand [8,9,10]. The blunt-ended telomeres have been proposed to be created from leading-strand synthesis and associate with the KU70/KU80 heterodimer. This heterodimer inhibits the excessive elongation of telomeres by telomerase and the formation of extrachromosomal telomeric circles, which seem to arise as a result of intra-telomeric recombination. Therefore, the KU70/KU80 heterodimer also protects Arabidopsis telomeres. However, it does not protect them against inter-telomeric recombination events leading to telomere fusions [9,11,12,13,14]. Thus, the KU70/KU80 heterodimer protects telomeres differently in mammals and plants.

It is not clear whether a shelterin-like complex caps Arabidopsis telomeres and prevents telomere fusions. Seventeen putative double-strand telomeric repeat binding factors that could play the roles of TRF1 and TRF2 have been described in Arabidopsis. Among those are proteins like TBP1 or TRB1-5 that bind double-strand telomeric repeats in vitro, associate with telomeric regions, control telomere length homeostasis, and/or interact with telomerase as well as with other telomeric proteins [15,16,17,18]. However, these telomeric repeat binding factors have not been shown to cap telomeres, which might be related to functional redundancy. Whereas Arabidopsis homologues to TIN2, TPP1 and RAP1 have not been described, three Arabidopsis POT1 proteins (POT1a, POT1b and POT1c) have been reported. However, it is not clear whether these proteins bind to single-strand telomeric repeats in vivo or function in telomere capping, although some of them influence telomerase activity [19,20,21,22,23]. Therefore, the existence of a protective shelterin-like complex in Arabidopsis remains to be established.

The existence of a capping CST complex has been clearly shown in Arabidopsis. This complex includes the proteins CTC1, TEN1 and STN1, associates with the single-strand telomeric repeats through CTC1, and protects them. Mutants altered in any of the three proteins of the complex have severe morphological alterations, reduced fertility, dramatically short telomeres and instability phenotypes, including elevated levels of extrachromosomal telomeric circles and a high frequency of telomere fusions. Therefore, the Arabidopsis CST complex protects telomeres from different types of recombination events [24,25,26,27].

Usually, telomere fusions are detected by cytological studies (FISH) or by PCR with subtelomeric primers followed by Southern blot and hybridization. Since both techniques require one hybridization step, which is often performed with fluorescent telomeric probes (FISH) or radioactivity-labelled telomeric probes (PCR), they allow the detection of fusions containing telomeric sequences but not of those lacking them [28,29]. To bypass this limitation, we have developed a novel PCR procedure to detect telomere fusions containing or lacking telomeric repeats in *Arabidopsis thaliana*. This procedure is based on the performance of nested PCRs with subtelomeric primers, does not require hybridization, and allows the detection of fusions between multiple telomeric regions. We have used it to analyze telomere fusions in WT and null *ctc1-2* mutant plants and have found that the mutant plants, but not the WT plants, exhibit telomere fusions. Our results agree with previously reported data and further support the widespread end-capping function of Arabidopsis CTC1 by showing that all the telomeric regions of chromosomes 1, 3 and 5 fuse among them.

## 2. Results

### 2.1. Design of a Nested PCR Procedure to Display Telomere Fusions

**Figure 1:**
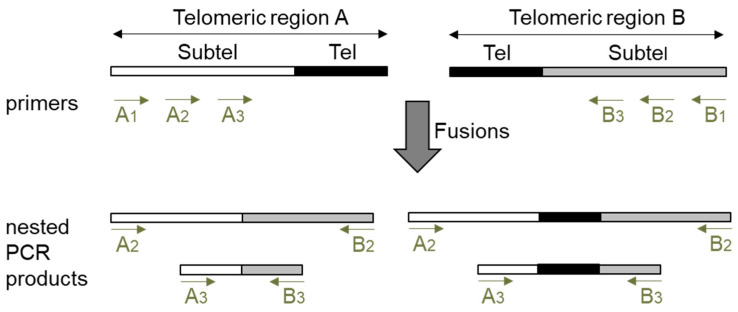
Design of a nested PCR procedure to detect telomere fusions. Schematic representation of two telomeric regions (A and B) and of the PCRs 2 and 3 products that are expected to be generated by the amplification of fusions between them. Telomeres (Tel) are represented by black filled rectangles and subtelomeres (Subtel) by white or grey filled rectangles. Whereas some PCRs 2 and 3 products could contain subtelomeric sequences and telomeric repeats, others might only contain subtelomeric sequences. A1, A2 and A3 refer to primers 1, 2 and 3 of telomeric region A (see text for a further explanation).

The detection of fusions between two specific telomeric regions by PCR has been traditionally performed with subtelomeric primers that direct DNA synthesis towards telomeres and by the further hybridization of the PCR products with a telomeric probe. This later step was required to avoid the detection of unspecifically amplified PCR products. When we planned to analyze telomere fusions by PCR, we also considered that unspecific PCR products should not be detected. However, since the hybridization step impairs the detection of fusion events lacking telomeric repeats, we decided to avoid it and bypass the specificity problem by performing three PCRs (PCR1, PCR2 and PCR3). The schematic representation of our PCR procedure is shown in Figure 1. We decided to design three subtelomeric primers (1, 2 and 3) for each telomeric region to be analyzed. Whereas primers 1 should be the most telomere-distal, primers 3 should generally be the most telomere-proximal. To detect fusion events between two specific telomeric regions (A and B), first, PCR1 should be performed with primers A_1_ and B_1_. Then, the products obtained by PCR1 should be amplified by performing two nested PCRs, PCRs 2 and 3, with primers A_2_, B_2,_ A_3,_ and B_3_. Generally, but not always, PCR2 should be performed with primers 2 and PCR3 with primers 3. Finally, the size of the products obtained by PCRs 2 and 3 should be measured after resolving them on agarose gels. If the size of the PCR2 products is shifted with regard to the size of the PCR3 products according to the relative subtelomeric positions of the corresponding primers, we interpret that they reveal true fusion events between telomeric regions A and B. This procedure allows the detection of fusion events containing or lacking telomeric repeats as long as the subtelomeric sequences that anneal with primers 2 and 3 have not been removed by nucleolytic degradation of the unprotected telomeres.

We used the nested PCR technique to analyze all possible fusions between the ends of Arabidopsis chromosomes 1, 3 and 5. Independently of the pairs of telomeric regions analyzed, we always used the same subtelomeric primers for each telomeric region. The DNA sequences of all the primers are shown in Table 1, together with their distances to telomeres. The specific combinations of primers used to perform PCRs 2 and 3 together with the expected size shifts of their PCR products are shown in Table 2.

### 2.2. The Analysis of ctc1-2 Mutant Plants Validates the Nested PCR Technique

To validate the nested PCR technique, we decided to analyze the presence of telomere fusions in WT and in null *ctc1-2* mutant plants. A previous report has shown that null Arabidopsis *ctc1* mutants have a severe telomere deprotection phenotype as a consequence of telomere capping defects, accompanied by developmental abnormalities, sterility, and poor seed germination rates [25]. Since null *ctc1* mutants had been shown to exhibit a high frequency of fusions events, most of which lack telomeric repeats and only contain subtelomeric sequences, *ctc1-2* was optimal to test the nested PCR technique [25,31].

**Figure 2:**
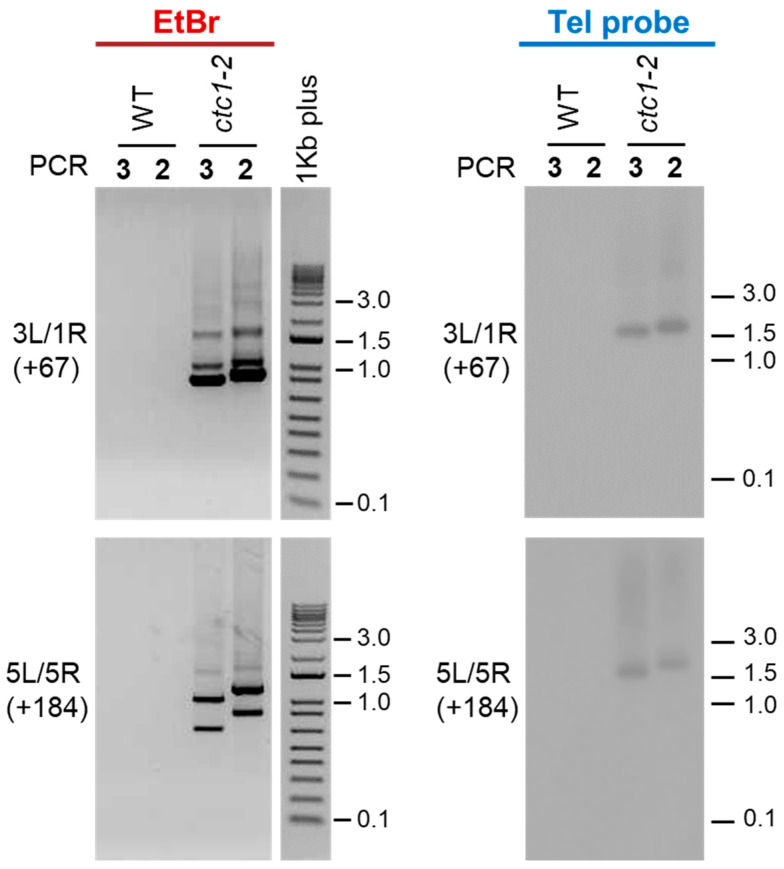
Detection of telomere fusions in WT and *ctc1-2* mutant plants. The PCR products obtained after performing PCRs 2 and 3 with the primers designed to analyze the fusions of 3L with 1R (3L/1R) and of 5L with 5R (5L/5R) are shown. The expected size shifts of PCR2 products with regard to PCR3 products are indicated between parentheses. The left panels display agarose gels containing PCR products stained with ethidium bromide (EtBr). The middle panels show 1Kb plus DNA ladders (Invitrogen) also stained with ethidium bromide and run in the same gels. The right panels display the same PCR products shown on the left panels but run on different agarose gels and hybridized with a telomeric probe (Tel probe).

We first analyzed fusions between the left telomeric region of chromosome 3 (3L) and the right telomeric region of chromosome 1 (1R) as well as between 5L and 5R. The expected size shifts of PCR2 with regard to PCR3 products for these combinations of telomeric regions are +67 and +184 bp, respectively. After running PCRs 2 and 3 on agarose gels and staining them with ethidium bromide, we did not observe PCR products using WT DNA. In contrast, smears and shifted PCR bands of the expected sizes were observed using *ctc1-2* DNA (Figure 2, left panels). Therefore, our nested PCR assay is clean because it does not detect PCR products using WT DNA and detects true telomere fusion events because the sizes of PCR products obtained with *ctc1-2* DNA are shifted as expected.

To differentiate between fusion events containing or lacking telomeric repeats, we decided to hybridize the 3L/1R and 5L/5R PCRs 2 and 3 products with a telomeric probe. Whereas no signals were detected after hybridization of the WT PCR products, smears and shifted bands of the expected sizes were detected after hybridization of the *ctc1-2* products (Figure 2, right panels). However, some of the *ctc1-2* PCR bands detected with ethidium bromide were not observed when the same bands were hybridized with the telomeric probe. Thus, our results are in agreement with previously reported data showing that most fusion events in *ctc1* mutants contain subtelomeric sequences but lack telomeric repeats [25,31].

### 2.3. Nested PCR Analyses Highlight the Widespread End-Capping Protection of Arabidopsis CTC1

**Figure 3:**
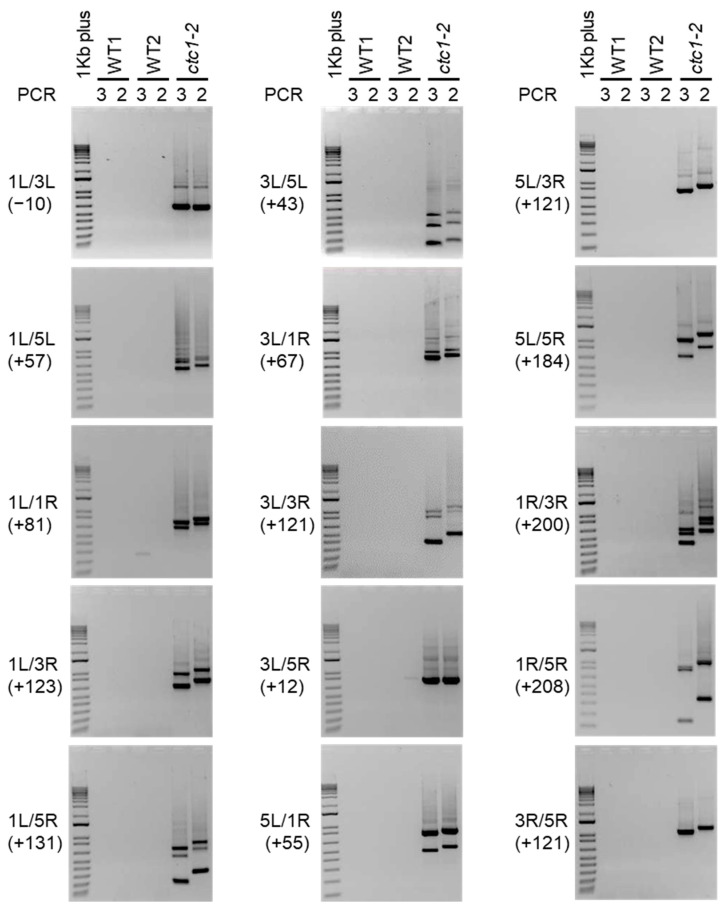
Analysis of telomere fusions between Arabidopsis chromosomes 1, 3 and 5. Panels in this figure show the ethidium bromide staining of the PCRs 2 and 3 products obtained after analyzing fusions between all possible combinations of 1L, 1R, 3L, 3R, 5L and 5R. A 1Kb plus DNA ladder is shown at the left of each panel. The telomeric regions involved in the fusions are indicated together with the expected size shifts of the corresponding PCR2 products with regard to PCR3 products (between parentheses). Fusions were analyzed in two independent groups of WT plants (WT1 and WT2) and in *ctc1-2* mutant plants. Note that the expected size shift for 1L/3L is negative due to the fact that primer 3L3 is more telomere-distal than primer 3L2 (see Table 1) and that primers 1L3 and 1L2 largely overlap.

To further analyze the instability phenotype of the *ctc1-2* mutant, we decided to study whether the rest of the telomeric regions present in chromosomes 1, 3 and 5 fuse among them. In general, we did not detect PCR products after ethidium bromide staining when amplifying WT DNA, although some PCR bands that were only observed in PCRs 2 or 3 or that did not shift as expected could be eventually amplified. In turn, we detected PCR products for all possible combinations of the telomeric regions when amplifying *ctc1-2* DNA (Figure 3). Since the sizes of the smears and PCR bands shifted according to the positions of the subtelomeric primers in all cases, we concluded that all the telomeric regions analyzed fuse among them in *ctc1-2*, which we have verified for specific combinations of telomeric regions by sequencing some of the corresponding PCR bands (Supplementary Figure S1). These results highlight the low background levels of the technique and support that telomere fusions in *ctc1-2* are not limited to specific combinations of telomeric regions.

## 3. Discussion

Telomere fusions are usually analyzed by FISH or PCR followed by Southern-blot and hybridization [28,29]. The FISH technique usually focuses on the observation of a few tens or hundreds of cells under a fluorescence microscope after hybridizing them with a fluorescent telomeric probe. If anaphase bridges containing telomeric sequences are detected, they are assumed to arise from the previous fusion of two telomeric regions. However, these bridges could also arise as a result of recombination events taking place at Interstitial Telomeric Sequences (ITSs), which can also hybridize with the telomeric probe. Although this limitation of the FISH technique could be bypassed by hybridizing with multiple subtelomeric sequences [25,31], it is common that the results obtained by FISH are verified by PCR. The detection of telomere fusions by PCR requires the use of subtelomeric oligos that prime DNA synthesis towards telomeres. These subtelomeric primers should only render PCR products arising from specific telomere fusion events. However, considering the high number of PCR cycles that should be performed to detect these events, the subtelomeric primers could also generate unspecific PCR products with low frequency. To avoid this specificity problem, the PCR products generated by the subtelomeric primers have been usually resolved on agarose gels and hybridized with a telomeric probe. In this way, only true telomeric fusion events are expected to be displayed. However, unspecifically amplified DNA fragments containing ITSs could also be detected. To bypass this limitation and detect specific telomere fusions containing or lacking telomeric sequences, we have developed a nested PCR technique.

We have validated the nested PCR technique by analyzing a *ctc1-2* mutant. As mentioned above, Arabidopsis *ctc1* mutants have been previously shown to have a severe telomere deprotection phenotype accompanied by developmental abnormalities, sterility and poor seed germination rates [25]. In addition, they exhibit a high frequency of telomere fusions, which have been detected by FISH and PCR. FISH experiments have shown extensive fusion events in *ctc1* mutants but have not revealed which telomeric regions fuse among them. This information has been partially provided by PCR experiments followed by hybridization with a telomeric probe, which have detected fusions between several pairs of telomeric regions [25,27,32,33,34]. These results are in full agreement with our studies, which show that null *ctc1-2* mutant plants display fusions between all possible combinations of the telomeric regions present in chromosomes 1, 3 and 5. Therefore, our results support that the loss of CTC1 could lead to fusion events involving any specific combination of Arabidopsis telomeric regions.

It has been previously proposed that whereas CTC1 and the CST complex cap all Arabidopsis chromosome ends containing 3′ overhangs, the KU70/KU80 heterodimer caps the blunt-ended telomeres [14]. However, FISH and PCR analyses have shown that the loss of KU70 or KU80 does not lead to telomere fusions, which should reflect the presence of additional capping mechanisms at blunt-ended telomeres [9,11,12,13,14]. Our results are in agreement with this proposal because they support the widespread end-capping protection achieved by CTC1. In addition, our unpublished nested PCR analyses verify that a *ku70* mutant does not undergo telomere fusions. 

The nested PCR technique described here is simple, because it only involves three PCR reactions, fast, because it can be achieved in hours, clean, because it has very low levels of background, and robust, because it allows the detection of specific telomere fusions between multiple telomeric regions. This technique can help to understand how telomere shortening, the loss of telomeric chromatin structure, or environmental aggressions influence telomere capping and genome stability in Arabidopsis. In addition, the nested PCR technique could be adapted to other organisms with sequenced genomes, including many other plant species. Therefore, it could contribute to the study of their telomeres biology by allowing the identification of their telomere capping proteins or the determination of the minimal functional length of their telomeres and its influence on cell and tissue viability.

## 4. Materials and Methods

### 4.1. Plant Materials and Growth Conditions

Wild-type *Arabidopsis thaliana* (Col-0) and homozygous G2 *ctc1-2* mutant plants were analyzed. Heterozygous *ctc1-2* seeds were obtained from the European Arabidopsis Stock Center (NASC) and segregated to obtain homozygous G1 plants. The *ctc1-2* mutant, which was generated by T-DNA insertion (SALK_114032), has been previously described [25]. Arabidopsis seeds were sterilized and plated on Murashige and Skoog (1× MS) medium (Duchefa Biochemie, Haarlem, The Netherlands) with 0.8% agar plus 1% sucrose and stratified for 3 days at 4 °C in darkness before being transferred to the growth chamber under long-day condition (16 h light/8 h dark, 23 °C). Then, after two weeks, about 25 seedlings were collected and used for genomic DNA isolation.

### 4.2. DNA Isolation and PCR Amplification

Genomic DNA was isolated using a CTAB-based method [35]. Essentially, plants were frozen on liquid nitrogen, ground, and incubated at 65 °C for 1 h in DNA extraction buffer (150 mM Tris pH 7.4; 1 M NaCl; 15 mM EDTA; 1.5% CTAB; 0.1% β-ME). Then, after chloroform extraction, DNA was precipitated with 0.6 volumes of isopropanol, RNase treated, phenolized, precipitated with ethanol, and resuspended in water [36].

PCR reactions (25 μL) contained 1x Immobuffer, 2.5 mM MgCl_2_, 200 μM dNTPs, 0.6 μM of each primer, 50–100 ng of DNA, and 0.625 U of Immolase DNA Polymerase (Bioline Meridian Bioscience, Memphis, TN, USA), which needs heat activation. PCR conditions were as follows: 95 °C × 10 min; 28 or 35 cycles at 94 °C × 15 s, 60 °C × 30 s and 72 °C × 1 min; 72 °C × 5 min. Since we decided to focus our telomere fusion analyses on the left (L) and right (R) telomeric regions of chromosomes 1, 3 and 5, we designed primers to amplify them. Three different subtelomeric primers (1, 2 and 3) were designed for each telomeric region. Whereas type 1 primers are most distal to telomeres, type 3 primers are most proximal (except for 3L) and all of them direct DNA synthesis towards telomeres. These primers were expected to generate PCR products by amplifying the junctions of fusions between the different telomeric regions (see Figure 1).

We decided to design primers that align at the centromeric side of subtelomeric heterochromatin, which extend about 1–2 kbp from Arabidopsis telomeres and, in some regions, share extensive DNA homology. This is the case for the subtelomeric ITSs, which abut or are very near to telomeres, and for other regions like 1R and 4R that have 93% of identity between coordinates 163–857 and 518–1258, respectively, according to TAIR10-Tel [30]; 5L and 5R that also share 93% of identity between coordinates 206–848 and 669–1321; or 1R, 3L and 5L between coordinates 864–1332, 1241–1645 and 966–1281, which share lower percentages of identity (about 65%). Since the primers designed align far from the telomere–subtelomere boundaries, they allow the detection of fusions containing or lacking telomeric sequences. We designed primers with similar melting temperatures and low probabilities of self-annealing or self-dimer formation. Those used in this study were selected according to their compatibility and specificity.

Three PCR reactions were performed to study the fusions between two specific telomeric regions. The first one (PCR1) involved amplifying 50 ng (*ctc1-2*) or 100 ng (WT) of genomic DNA with primers 1 during 28 cycles. The second and third ones (PCRs 2 and 3) involved primers 2 and 3 (see Figure 1). PCRs 2 and 3 were performed using 1 μL of a 1/100 dilution of PCR1 during 35 cycles. The sequences of all the primers used are listed in Table 1 and their combinations to perform the nested PCRs 2 and 3 are listed in Table 2. Two μL (*ctc1-2*) or 10 μL (WT) of PCRs 2 and 3 were resolved on 1% agarose gels and visualized with Ethidium Bromide. Then, the size of the PCR products was measured using 1Kb plus DNA ladders (Invitrogen Thermo Fisher Scientific, Vilnius, Lithuania) as reference. We interpreted that these PCR products reveal telomere fusions if the size of the PCR2 products exhibited the expected shift with regard to the size of the PCR3 products according to the location of the primers (see Table 2).

### 4.3. DNA Hybridization

PCRs 2 and 3 corresponding to the analyses of fusions between 3L and 1R and between 5L and 5R were run on 1% agarose gels, transferred to Hybond^TM^-XL membranes (GE Healthcare, Buckinghamshire, UK), and hybridized with a ^32^P-labeled telomeric probe. Whereas 10 μL of WT PCR products were run on the gel, only 2 μL of a 1:500 dilution were loaded for *ctc1-2*. The telomeric template used for hybridization was constructed by annealing and ligation of telomeric sequence oligos containing specific restriction sites at their ends. These sites were used for cloning in pUC18. The sequence of the template is as follows: HindIII site–(TTTAGGG)_12_-SphI site–(TTTAGGG)_10_–SalI site-(TTTAGGG)_15_–EcoRI site. Prior to hybridization, the template was excised from pUC18 using the HindIII and EcoRI sites, purified and labeled using random hexanucleotides primers and Klenow, as indicated by the manufacturer (Roche Diagnostics, Mannheim, Germany). Hybridizations were performed at 65 °C overnight in hybe solution (5× SSPE, 5× Denhardt’s, 0.5% SDS and 40 μg/mL of denatured salmon sperm DNA). Filters were washed twice in 2× SSPE plus 0.1% SDS for 10 min at 65 °C and once in 1× SSPE plus 0.1% SDS for 30 min at 65 °C. Hybridization signals were detected using the Kodak Biomax transcreen-HE (Eastman Kodak, Rochester, NY, USA) and measured using 1Kb plus DNA ladders that were run in the gels but not transferred to the membranes [36].

## 5. Conclusions

Telomeres protect the ends of linear eukaryotic chromosomes from being recognized as DNA double strand breaks. Two major protein complexes are involved in the protection of telomeres: shelterin and CST. The dysfunction of these complexes can generate genomic instability and lead to telomere fusions, which are catastrophic for the cells. Therefore, monitoring telomere fusions helps to understand telomeres biology. Telomere fusions are often detected by cytological studies (FISH) or by PCR with subtelomeric primers. Usually, both methods involve hybridization with a telomeric probe, which allows the detection of fusions containing telomeric sequences but not of those lacking them. To bypass this limitation, we have developed a novel PCR procedure that allows the detection of telomere fusions containing or lacking telomeric repeats in *Arabidopsis thaliana*. This procedure is based on the performance of nested PCRs with subtelomeric primers and does not require hybridization. We have used it to analyze telomere fusions in wild-type and mutant plants altered in CTC1, one of the three components of the Arabidopsis CST telomere capping complex. Our results validate the nested PCR method and highlight the widespread end-capping protection achieved by CTC1. We envision that this nested PCR method will become a valuable tool to study telomere biology. It could help to identify telomere capping proteins or to determine the influence of environmental conditions and telomere length on genomic stability in Arabidopsis and in many other organisms.

## Figures and Tables

**Table 1:** Primers used for PCR amplification. The DNA sequences of the primers designed for the left (L) and right (R) telomeric regions of Arabidopsis chromosomes 1, 3 and 5 are shown. Whereas primers 1 are the most telomere-distal, primers 3 are the most telomere-proximal, except for 3L. The labels 1L_1_, 1L_2_ and 1L_3_ refer to primers 1, 2 and 3 for the left telomeric region of chromosome 1. The distances of the 5′ ends of the primers to the telomere–subtelomere boundaries according to the TAIR10-Tel version of the Arabidopsis genome are indicated [30].

Telomeric Region	Primer	DNA Sequence (5′–3′)	Distance to Telomere (bp)
1L	1L1	CATGGAGGAATCACAGAACTATCA	1373
1L	1L2	TTGCCACTTTCTGCTTCACAA	1194
1L	1L3	GCCACTTTCTGCTTCACAAGTT	1192
1R	1R1	GAATCTTGTGAGTGATGGAAGCT	1406
1R	1R2	ACCACAATATGCCAGCTGTATC	1376
1R	1R3	TGCACTTCTTCAGACTTAGTTATCC	1297
3L	3L1	GCAAGATTTGGGTTTCCTCGTA	1766
3L	3L2	GAAGAAAGCAAGGTATATATTCTGATGA	1700
3L	3L3	AGATAAAACAAAGAAGAAAGCAAGGT	1712
3R	3R1	GCTTAGTTGCTTTCCCGACAA	2205
3R	3R2	CTCAACTCCTTTAGTGCTAATTG	2184
3R	3R3	ACGTACTCTTGTTACTTGCGTC	2063
5L	5L1	ACTCTGAACCCTTTGAAATACATCA	1381
5L	5L2	ATATCTGCTACCAGGAACATAC	1345
5L	5L3	ATTGCAGAAGGTGAAAATTGGC	1290
5R	5R1	ACGGCTACCTCAAGAAAATGC	2233
5R	5R2	ATGTAGATAGATAACTCATCCTCATT	2209
5R	5R3	TCGTGAAATCATCCCCAAATCG	2080

**Table 2:** Combinations of primers used to detect telomere fusions. The specific combinations of primers used to detect fusions between the different telomeric regions are shown. The label 1L/3L refers to the analysis of fusions between 1L and 3L and so on. For each combination of telomere fusions analysis, the expected size shift (bp) of the PCR2 products with regard to the PCR3 products is also shown. The negative value corresponding to 1L/3L indicates that a small decrease in the size of the PCR products is expected.

Telomere Fusion	PCR1 Primers	PCR2 Primers	PCR3 Primers	Expected Shift (bp)
1L/3L	1L1 + 3L1	1L2 + 3L2	1L3 + 3L3	−10
1L/5L	1L1 + 5L1	1L2 + 5L2	1L3 + 5L3	+57
1L/1R	1L1 + 1R1	1L2 + 1R2	1L3 + 1R3	+81
1L/3R	1L1 + 3R1	1L2 + 3R2	1L3 + 3R3	+123
1L/5R	1L1 + 5R1	1L2 + 5R2	1L3 + 5R3	+131
3L/5L	3L1 + 5L1	3L2 + 5L2	3L3 + 5L3	+43
3L/1R	3L1 + 1R1	3L2 + 1R2	3L3 + 1R3	+67
3L/3R	3L1 + 3R1	3L2 + 3R2	3L2 + 3R3	+121
3L/5R	3L1 + 5R1	3L3 + 5R3	3L2 + 5R3	+12
5L/1R	5L1 + 1R1	5L2 + 1R2	5L3 + 1R2	+55
5L/3R	5L1 + 3R1	5L2 + 3R2	5L2 + 3R3	+121
5L/5R	5L1 + 5R1	5L2 + 5R2	5L3 + 5R3	+184
1R/3R	1R1 + 3R1	1R2 + 3R2	1R3 + 3R3	+200
1R/5R	1R1 + 5R1	1R2 + 5R2	1R3 + 5R3	+208
3R/5R	3R1 + 5R1	3R2 + 5R3	3R3 + 5R3	+121

## Data Availability

Data are contained within the article and Supplementary Materials.

## Supplementary Materials

The following supporting information can be downloaded at: https://www.mdpi.com/article/10.3390/ijms25010672/s1.
