# Supplementary material for: A Nested PCR Telomere Fusion Assay Highlights the Widespread End-Capping Protection of Arabidopsis CTC1
Source: Int J Mol Sci. 2024 Jan 4;25(1):672. doi: 10.3390/ijms25010672 (PMC10779545; doi:10.3390/ijms25010672)
Supplement: Supplementary file 1 [file ijms-25-00672-s001.zip › ijms-2700738-supplementary.pdf]

A

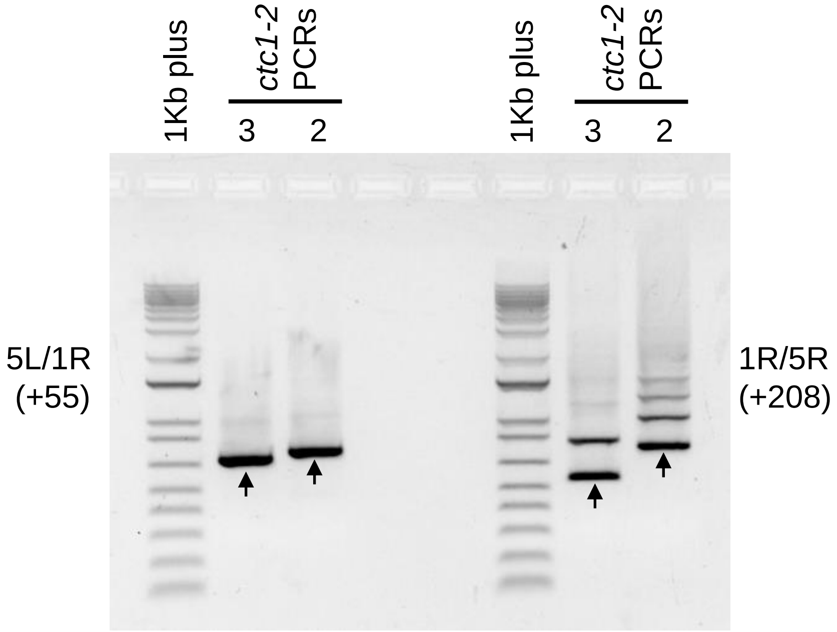

B

DNA sequence of the 5L/1R purified bands

atatctgctaccaggaacatactattttgtttgcatttacttcaatttcaatatcattgcagaaggtgaaaattggcaaagaggagataa
cgggccgtctaaataggccggtgttgatgctaatgggcccattgcggctggaaaatattaaactaagtagacaagaaggaattaaaatga
ggagggttgttcttatattgatattttcacaaggatagttgttacaagacgcaaaaatacaaatgtccaaacaaggaaaactaaagccaa
cagacaaaataaaagtcctcaaaaaatagaaggtttatagcgtcctctttcgattcgacttttctatttggtatttatgatgtgtttagc
ttccctttcatagggcttagtttgcgagaaaatcacgtcttccgccggccttagtttttggatatttttatttcgtccgccgacttaagt
tttctttgtttaaacatttgtatttctctttagtaccacaacaagtttaattaatcaaatttcagatgagaaaaaaaatgttatgtgcgt
gttgcgtcttgtaacaactatacttgtgaaaatataagaacaatccctcttcattttaattccttcctggctacttagtttaatatcttc
cagccgggataactaagtctgaagaagtgcagaaaactgagtctttttcagcgatgtattcgccaattccccttctttatttcagcaga
tacagctggcatattgtggt

DNA sequence of the 1R/5R purified bands

accacaatatgccagctgtatctgctgaaataaagaaggggaattggcgaatacatcgctgaaaaagactcagttttctgcacttcttc
agacttagttatcccggctggaagatattaaactaagtagccaggaaggaattaaaatgaagagggattgttcttatattttcacaagta
tagttgttacaagacgcaacacgcacataacatttttttctcatctgaaatttgattaattaaacttgttgtggtactaaagagaaata
caaatgtttaaacaaagaaaacttaagtcggcggacgaaataaaaatatccaacctccatggtggaatctatggtggaatctctagtgat
tcgttactagattggcttgtggagattgaagaaatttgggagtttcaaaacgtttcggatgatctatttgtttcgcttgtagttaagtaa
tttcgagggcaagccgcatcatggtggcaacaagtacagaaaacaaacaaggcaagaatcctattaaatcatgggataaacttaaggaga
agctatgcaaaactttttcacctcctcattatacgagtttggtgtctaatcgtttgaagaattaaaacaaagagcccgatttggggatga
tttcacgaagaattgtcacgttcaagtcaacaacttcgtggagaagcctgcttatgatcagatggtcacgttcaagacaaggactttgtt
gctaagtccgtccatggtattaatgaggatgagttatctatctacat

Supplementary Figure S1. Sequencing of *ctc1-2* fusion products. (**A**) Ethidium bromide stained *ctc1-2* PCR products obtained after performing PCRs 2 and 3 with the primers designed to analyse the fusions of 5L/1R (left) and of 1R/5R (right). Kb plus ladders are shown at both sides of the panel. The expected size shifts of PCR2 products with regard to PCR3 products are indicated between parenthesis. Arrows point to the PCR bands that were purified and sequenced. (**B**) DNA sequences of the purified PCR bands. Not underlined letters correspond to the sequences of PRC3 bands. PRC2 bands contain the sequences underlined and not underlined. Note that the size shifts detected by sequencing are those expected. Whereas the sequences of 5L are in red, the sequences of 1R are in blue and the sequences of 5R are in brown. The black sequence in the junction of 1R/5R does not correspond to any of the telomeric regions. The sequences corresponding to the primers used for amplification and sequencing are highlighted in yellow.
